# Frequency-Specific Functional Connectivity Density as an Effective Biomarker for Adolescent Generalized Anxiety Disorder

**DOI:** 10.3389/fnhum.2017.00549

**Published:** 2017-12-05

**Authors:** Zhe Zhang, Mei Liao, Zhijun Yao, Bin Hu, Yuanwei Xie, Weihao Zheng, Tao Hu, Yu Zhao, Fan Yang, Yan Zhang, Linyan Su, Lingjiang Li, Jürg Gutknecht, Dennis Majoe

**Affiliations:** ^1^Gansu Provincial Key Laboratory of Wearable Computing, School of Information Science and Engineering, Lanzhou University, Lanzhou, China; ^2^Mental Health Institute of the Second Xiangya Hospital, Central South University, Changsha, China; ^3^The China National Clinical Research Center for Mental Health Disorders, National Technology Institute of Psychiatry, Key Laboratory of Psychiatry and Mental Health of Hunan Province, Changsha, China; ^4^CAS Center for Excellence in Brain Science and Intelligence Technology, Shanghai Institutes for Biological Sciences, Chinese Academy of Sciences, Shanghai, China; ^5^Guangdong Mental Health Center, Guangdong General Hospital, Guangzhou, China; ^6^Computer Systems Institute, ETH Zürich, Zürich, Switzerland

**Keywords:** adolescent generalized anxiety disorder, frequency-specific, functional connectivity density, CEEMDAN, classification

## Abstract

Several neuropsychiatric diseases have been found to influence the frequency-specific spontaneous functional brain organization (SFBO) in resting state, demonstrating that the abnormal brain activities of different frequency bands are associated with various physiological and psychological dysfunctions. However, little is known about the frequency specificities of SFBO in adolescent generalized anxiety disorder (GAD). Here, a novel complete ensemble empirical mode decomposition with adaptive noise method was applied to decompose the time series of each voxel across all participants (31 adolescent patients with GAD and 28 matched healthy controls; HCs) into four frequency-specific bands with distinct intrinsic oscillation. The functional connectivity density (FCD) of different scales (short-range and long-range) was calculated to quantify the SFBO changes related to GAD within each above frequency-specific band and the conventional frequency band (0.01–0.08 Hz). Support vector machine classifier was further used to examine the discriminative ability of the frequency-specific FCD values. The results showed that adolescent GAD patients exhibited abnormal alterations of both short-range and long-range FCD (S-FCD and L-FCD) in widespread brain regions across three frequency-specific bands. Positive correlation between the State Anxiety Inventory (SAI) score and increased L-FCD in the fusiform gyrus in the conventional frequency band was found in adolescents with GAD. Both S-FCD and L-FCD in the insula in the lower frequency band (0.02–0.036 Hz) had the highest classification performance compared to all other brain regions with inter-group difference. Furthermore, a satisfactory classification performance was achieved by combining the discrepant S-FCD and L-FCD values in all frequency bands, with the area under the curve (AUC) value of 0.9414 and the corresponding sensitivity, specificity, and accuracy of 87.15, 92.92, and 89.83%, respectively. This study indicates that the alterations of SFBO in adolescent GAD are frequency dependence and the frequency-specific FCD can potentially serve as a valuable biomarker in discriminating GAD patients from HCs. These findings may provide new insights into the pathophysiological mechanisms of adolescent GAD.

## Introduction

Generalized anxiety disorder (GAD), as a common subtype of anxiety disorder, is a prevalent psychiatric disease which often occurs in adolescence or early adulthood, characterized by frequently excessive as well as uncontrollable fear and worry about a variety of things such as school life, social work, and individual ability (Beesdo et al., 2010). Moreover, GAD patients are often easily fatigued, restless, and difficult to concentrate or blank in mind with symptoms of muscle tension, thus contributing to distress and functional impairment, which severely affects patients’ quality of life and to some extent increases social and economic burden (Kessler et al., 2005; Hoffman et al., 2008). To make things worse, GAD patients spend more time on treatment due to lower disease recognition rate and higher degree of differentiation compared with other types of anxiety disorder (Chavira et al., 2004). During the past decades, although a vast number of studies have focused on exploring GAD as well as adolescents with GAD, unfortunately, the pathophysiological mechanisms of GAD still remain poorly understood.

Resting-state functional connectivity (FC) that measures the temporal coherence of blood oxygen level-dependent (BOLD) signals between two spatially distinct regions has been widely applied to explore the spontaneous functional brain organization (SFBO) of GAD. Previous GAD studies have focused largely on amygdala-based connectivity and showed aberrant amygdala connectivity with several brain regions including prefrontal cortex (Monk et al., 2008; Etkin et al., 2009), anterior cingulate cortices (Etkin et al., 2010; Blair et al., 2012), insula (Hamm et al., 2014), and limbic system (Roy et al., 2013; Liu et al., 2015c), which indicates that the amygdala may play an important role in functional neural circuitry underlying the progression of GAD symptomatology (Hilbert et al., 2014; Makovac et al., 2016). Recently, a few studies have extended the investigation of abnormal FC of GAD patients to more brain regions. The alteration of FC in GAD patients has been found between prefrontal and limbic brain regions that may be related to emotional regulation deficits in GAD (Wang W. et al., 2016). However, all of the above-mentioned resting-state FC studies of GAD have mainly focused on BOLD signals within a broad frequency band (0.01–0.08 Hz), so some meaningful information related to specific frequency bands underling GAD may be missing.

It is widely accepted that human brain is a complex biological system in which a considerable number of oscillatory waves work together. With regard to the signals within different frequency bands, they may come from distinct oscillators with specific neural processes (Buzsa and Draguhn, 2004; Athanasiou et al., 2016) and physiological functions (Knyazev, 2007). Therefore, subdivision of the frequency band (0.01–0.08 Hz) may be an efficient approach in exploring specific functional activities in both healthy controls (HCs) and patients with mental disorders. For example, by carrying out a whole-brain amplitude low-frequency fluctuation (ALFF) analysis, a resting-state fMRI study observed the spontaneous activity at four different frequency bands (slow-5: 0.01–0.027 Hz, slow-4: 0.027–0.073 Hz, slow-3: 0.073–0.198 Hz, and slow-2: 0.198–0.25 Hz) in healthy human brain. Specifically, the activities in slow-5 were more active on cortical structures, whereas activities in slow-4 decreased in the subcortical structures, suggesting that the spontaneous brain activities were frequency-dependent (Zuo et al., 2010). Additionally, frequency-specific changes of SFBO have also been found in several psychiatric and neurological diseases, such as depression (Wang L. et al., 2016), social anxiety disorder (Zhang et al., 2015), epilepsy (Wang et al., 2015), Parkinson’s disease (Qian et al., 2016), mild cognitive impairment (Han et al., 2011), internet gaming disorder (Lin et al., 2015), migraine (Farago et al., 2017), and Wilson’s disease (Hu et al., 2017). To date, little is known about the frequency specificities of SFBO in adolescent GAD.

Moreover, the majority of research on GAD has revealed aberrant FC concentrated on seed-based analyses or predefined networks, which may neglect some unpredictable findings, because neither seed-based analyses nor predefined networks can effectively measure the FCs of the whole-brain. Recently, functional connectivity density (FCD) mapping, a novel voxel-wise FC method, has been developed to detect SFBO by measuring the local centrality of every voxel in the whole-brain connection (Tomasi and Volkow, 2010; Tomasi et al., 2016). Unlike the seed-based FC approach, FCD is a data-driven method, the requirement of no *priori*-knowledge making it more proper for exploratory analyses (Tomasi et al., 2016). In general, FCD reflects the complexity of the connectome as a whole and can fully exhibit the altered cortical and subcortical functional hubs underlying SFBO. According to the neighboring relationship between two brain voxels, FCD can be further divided into short-range FCD (S-FCD, the FC in local regions) and long-range FCD (L-FCD, the subtraction of FC between in the whole-brain and a local region) (Tomasi and Volkow, 2012), which can be served as indicators to explore abnormal functional organization in blind participants (Qin et al., 2015), post-traumatic stress disorder (Zhang et al., 2016), male heavy drinkers (Shokri-Kojori et al., 2016), irritable bowel syndrome (Weng et al., 2016), generalized tonic-clonic seizures (Zhu et al., 2016), and adolescents with pure conduct disorder (Lu et al., 2017).

So far, the mechanism of GAD-related alterations in frequency-specific FCD remains unclear. The present study aims to investigate how GAD affects the S-FCD and L-FCD in adolescents across different frequency bands. The complete ensemble empirical mode decomposition with adaptive noise (CEEMDAN) was applied to decompose the BOLD signals into four different frequency bands, and the S-FCD and L-FCD in each frequency band were calculated in further analysis. Additionally, we also aim to examine whether the frequency-specific FCD could help to discriminate patients with GAD from HCs. Specifically, support vector machine (SVM) with linear kernel was performed in discrimination between the patients and controls. Previous findings have revealed whole-brain FC had a good diagnostic potential for anxiety disorder (Liu et al., 2015a). Moreover, FC changes would reflect alterations in GAD symptomatology and can be as a treatment-relevant biomarker of symptom expression (Makovac et al., 2016). Therefore, we hypothesized that frequency-specific FCD could be used as a potential biomarker to distinguish patients from controls.

## Materials and Methods

### Participants

A total of 59 participants were recruited from local high schools in Hunan Province, including 31 GAD patients and 28 age- and sex- matched HCs. All the participants were enrolled via advertisements and school notice, which have been demonstrated in our previous study (Liao et al., 2013, 2014). Firstly, 1885 subjects finished the Screen for Child Anxiety Related Emotional Disorders (SCARED) which is a 41-item self-report questionnaire. The SCARED turns out to be a reliable and valid screening tool for childhood anxiety disorder, and it has an optimal total cutoff point score of 25 to separate children who are with anxiety disorders from those without (Su et al., 2008). Among 1885 subjects, 508 subjects’ SCARED scores were higher than or equal to 25, while 1377 subjects’ SCARED scores were lower than 25. Then, 673 subjects (508 SCARED scores ≥ 25; 165 SCARED scores < 25) were investigated by the same trained clinician and diagnosed using DSM-IV criteria and the Schedule for Affective Disorders and Schizophrenia for School Age Children-Present and Lifetime (K-SADS-PL) version (Lauth et al., 2010). To assess clinical activity, all participants performed the State-Trait Anxiety Inventory (STAI) (Spielberger et al., 1970), the Penn State Worry Questionnaire (PSWQ) (Meyer et al., 1990), and the Beck Depression Inventory (BDI) (Beck and Beamesderfer, 1974) before the fMRI scan. STAI is a self-administered evaluation system to assess anxiety severity, such as trait anxiety and state anxiety. PSWQ, consisting of 16 self-report items, is considered as a reliable scale to evaluate worry frequency and intensity, and with good test-retest reliability and enabling to distinguish GAD patients from patients with other anxiety disorders. BDI, a rating inventory containing 21 self-report items, can assess characteristic attitudes and depression symptoms, and shows high internal consistency in psychiatric populations and non-psychiatric, respectively. Specifically, participants with current first episode GAD without co-morbidity disorders and HCs without mental disorders and physical disease were included, whereas participants with history of seizures, neurological abnormalities, head trauma or unconsciousness, physical disease, and use of psychoactive substances were excluded. All participants were ensured to be non-medicated, right-handed, and voluntary. Each participant and one of his or her legal guardians were given written informed consent before the study. This study was approved by the local medical ethics committee at the Second Xiangya Hospital of Central South University. The demographic and neuropsychological characteristics of all the participants are detailed in **Table 1**.

**Table 1:** Demographics and neuropsychological characteristics of the participants.

Characteristics	GAD (*n* = 31)	HC (*n* = 28)	*P*-value
Characteristics	(*Mean* ± *SD*)	(*Mean* ± *SD*)	
Age (years)	16.90 ± 0.12	16.46 ± 0.18	0.44^a^
Handedness (right/left)	31/0	28/0	0.99^b^
Gender (males/females)	14/17	16/12	0.36^b^
IQ	102.7 ± 7.86	105.4 ± 9.20	0.19^a^
STAI			
State	43.48 ± 8.51	39.86 ± 7.87	0.02^a^
Trait	52.34 ± 7.63	44.25 ± 9.18	0.00^a^
BDI	8.36 ± 4.60	6.12 ± 5.21	0.10^a^
PSWQ	55.16 ± 9.76	38.79 ± 11.26	0.00^a^


*Values represented *mean* ± *SD*. SD, standard deviation; IQ, intelligence quotient; STAI, State-trait Anxiety Inventory; BDI, Beck Depression Inventory; PSWQ, Penn State Worry Questionnaire; GAD, generalized anxiety disorder; HC, healthy control. ^a^The *P*-value were obtained by two-sample *t*-tests. ^b^The *P*-value were obtained by Chi square test.*

### Data Acquisition and Preprocessing

All data were collected using a 3.0 Tesla Philips scanner equipped with a SENSE-8 channel head coil at the Second Xiangya Hospital of Central South University, Hunan, China. Before the scan, participants were instructed to lie relaxed, keep eyes closed, stay awake, think of nothing in particular, and move as little as possible during scanning. Head motion and scanner noise were controlled by foam paddings and earplugs. After fMRI scan, each participant was given a questionnaire to ensure whether he/she had fallen asleep or opened eyes during the time of scanning and no participant’s data was excluded. High-resolution T1-weighted anatomical images were acquired for registration in the sagittal orientation using a magnetization-prepared rapid gradient-echo sequence (repetition time = 7.5 ms, echo time = 3.7 ms, slice thickness = 1 mm, field of view = 256 mm × 256 mm, flip angle = 8°, and 180 transverse slices covered the whole brain). Resting-state fMRI data were acquired using a single-shot, gradient-recalled echo planar imaging sequence (repetition time = 3000 ms, echo time = 30 ms, slice thickness = 4 mm, slice gap = 0.38 mm, field of view = 240 mm × 240 mm, in-plane matrix = 64 × 64, flip angle = 90°, and 36 slices covered the whole brain) to measure 200 functional volumes for each subject in a total scan time of 600 seconds.

The data preprocessing was performed using DPARSF^1^ based on SPM12^2^. The main preprocessing steps included: (1) the first 10 functional images were discarded to stabilize the signal and adapt to inherent scanner noise. (2) The remaining functional images were corrected by slice-timing calibrating and head motion realigning. Specifically, they were first corrected differences in within-scan acquisition time among slices and then were realigned to the first volume for the purpose of correcting inter-scan head motion. Any data affected by head motion (maximal motion between volumes in each direction is greater than 3 mm, and rotation in each axis is greater than 3°) were discarded. We also calculated the mean frame-wise displacement (FD) of each participant on the basis of realignment parameters and the FD of each participant was less than 0.3 mm. There was no significant difference in FD (GAD: 0.12 ± 0.011; HC: 0.13 ± 0.013; *P* = 0.47) between two groups using two-sample *t*-test. (3) Functional images were spatially normalized to a standard three-dimensional space with the Montreal Neurological Institute (MNI) template and resampled to a resolution of 3 mm × 3 mm × 3 mm. To be more exact, a rigid-body transformation was used to co-register the individuals’ the high resolution structural images to the mean functional images. Then, all the co-registered images were segmented into gray matter, white matter and cerebrospinal fluid in MNI space by using “New Segment+DARTEL” in DPARSF. We applied the DARTEL procedure (Ashburner, 2007) to generate a study-specific template. (4) Linearly detrending and low-pass filtering (0–0.08 Hz) were performed to discard signal drifts caused by scanner instability or other sources. Particularly, we selected low-pass filtering rather than band-pass filtering (0.01–0.08 Hz) because previous studies have investigated brain activity within the frequency band of 0–0.01 Hz (Lin et al., 2015; Wang et al., 2015). (5) Nuisance covariates including head motion parameters, global mean signal, white matter signal and cerebrospinal signal were regressed out from the BOLD signals. Here, we removed global mean signals because, according to previous studies, the global signal removal can reduce physiological noise and improve the reliability of resting-state fMRI (Yan et al., 2013).

### Definition of Frequency of Interest (FOI)

After data preprocessing, a CEEMDAN method was applied to decompose the BOLD signals of each voxel into a finite set of intrinsic oscillatory components called intrinsic mode functions (IMFs), and each IMF occupies a unique frequency band (Colominas et al., 2014). CEEMDAN was developed from empirical mode decomposition (EMD) (Huang et al., 1998), which automatically decomposes non-stationary signals in a data-driven manner without any rigid predefined band-pass filter. However, the disadvantage of EMD would easily result in the “mode mixing” phenomenon, which always produces oscillations with very disparate frequencies in one IMF, or oscillations with similar frequencies in different IMFs. Also, the IMFs of EMD might contain some residual noise. CEEMDAN is a significant improvement on EMD, which are able to solve the problem of mode mixing and eliminate the residue noise in each IMF. For a given time series *x*(*t*), the CEEMDAN aims to extract a set of IMFs (*IMF_i_*(*t*), *i* = 1, 2,..., *K*) and a monotonic residue signal *r*(*t*), so that $x(t)=\Sigma_{i=1}^{k}{IMF}_{i}(t)+r(t)$. In this equation, the *t*, *i*, *K* denote the length of scanning time, the order of IMF, the number of IMF, respectively. CEEMDAN adopts an iterative method known as the sifting algorithm based on EMD to extract IMFs. The algorithm includes following steps: (1) Obtain the first residue by EMD. (2) Calculate the first IMF by subtracting the first residue from the raw signal. (3) Estimate the second residue and define the second IMF. (4) Repeat these steps until the last IMF is obtained. The detailed algorithm is presented in literature (Colominas et al., 2014). As a result of CEEMDAN, each IMF component occupies a distinctive frequency band: the first IMF occupies the highest frequency band, while the last IMF occupies the lowest frequency band, with the remaining ones in between, respectively.

After decomposition, the Hilbert weighted frequency (HWF) was applied to reflect the mean oscillation frequency of an IMF through instantaneous spectra information about amplitude and phase (Xie and Wang, 2006; Song et al., 2014). A brief computing step of HWF includes: (1) Compute Hilbert transform for each IMF. (2) Calculate the corresponding analytical signal. (3) Compute the HWF of each IMF with all data points. The detailed computing steps can be found in literature (Qian et al., 2016). Then we calculated all HWFs corresponding to IMFs in the whole brain gray matter across all participants and obtained the HWF distribution histograms in each group. In order to identify isolated frequency bands and lower the influence of extreme values, we used each corresponding HWF distribution component in 95% confidence intervals to derive a frequency of interest (FOI). Hence, the frequency band for each FOI would not overlap and can be considered independent from each other. To ensure the consistency of FOI between two groups, we chose maximum of lower limit and minimum of upper limit within each group frequency bands identified by 95% confidence interval to be lower and higher limiting value of final FOI, respectively. Besides, for comparison purposes, conventional frequency band from 0.01 to 0.08 Hz was selected as normal frequency of interest (FOI-N).

### Frequency-Specific FCD Mapping

The FCD mapping was generated by computing the number of functional connections of each voxel with the rest voxels in the whole-brain (Tomasi and Volkow, 2010). Usually, the FCD was divided, according to the functional connection properties (for example the length of connectivity), to short-range FCD (S-FCD) and long-range FCD (L-FCD). The S-FCD reflects the FC between voxels within a local cluster (intraregional), while L-FCD reflects the FC between a voxel within a local and the other without the local cluster (interregional) (Tomasi and Volkow, 2012). For a given voxel *x*_0_, its local cluster was determined by using a fast searching algorithm. Mathematically, the S-FCD value of a voxel *x*_0_ is computed as the numbers of functional connections above a correlation threshold between voxel *x*_0_ and the other voxels belong to the local cluster. This calculation is repeated for all voxels within whole-brain gray matter. Here, Pearson correlation analysis was introduced to evaluate the functional connection between a pair of voxels, and a correlation threshold above 0.6 was considered functionally connected, which proposed to be the optimal threshold for calculating FCD in previous studies (Zhang et al., 2016; Zhu et al., 2016). Similarly, the L-FCD equated to the numbers of functional connections between the voxel *x*_0_ within a local cluster and all other voxels without the local cluster in the whole-brain gray matter. The detailed computing procedure about S-FCD and L-FCD can be found in Tomasi and Volkow (2012).

The S-FCD and L-FCD within each predefined FOI of all participants were standardized by converting into *z*-scores. Then, the *z*-scored FCD maps were spatially smoothed with a full-width at half-maximum (FWHM) Gaussian kernel of 6 mm to minimize the differences in the functional anatomy of the brain across participants.

### Statistical and Classification Analyses

Two-sample *t*-tests and chi-square tests were implemented to investigate the group differences in continuous and categorical variables between the GAD patients and HCs by using SPSS21, respectively. Voxel-based comparisons of FCD mappings within the whole-brain gray matter mask were performed using two-sample *t*-tests with the DPABI software^3^. The resulting statistical maps were set at a corrected *P* < 0.05, with a cluster size of at least 220 voxels above an uncorrected *P* < 0.001, with estimated FWHM (14.22, 15.91, 15.69), performed by the AlphaSim program. Pearson correlation was conducted between FCD value and clinical questionnaires (STAI, BDI, and PSWQ) score of GAD patients in brain regions with significant group differences, with age and gender as covariates to be regressed out. Values with *P* < 0.05 (FDR corrected) were considered to indicate statistical significance.

**FIGURE 1:**
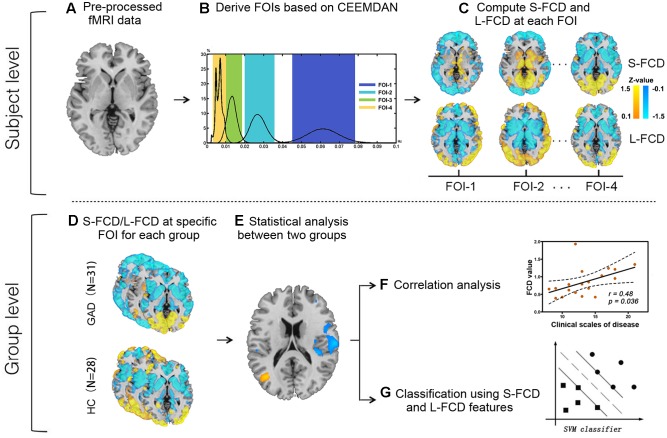
Flow diagram illustrating the proposed research framework. CEEMDAN, complete ensemble empirical mode decomposition with adaptive noise; S-FCD, short-range functional connectivity density; L-FCD, long-range functional connectivity density; FOI, frequency of interest; GAD, generalized anxiety disorder; HC, healthy control.

We further investigated whether the frequency-specific S-FCD/L-FCD with significant inter-group differences could discriminate the GAD patients from the HCs by means of the SVM classifier. SVM is a popular classifier and well-performed supervised learning model which works by projecting the low-dimensional non-separable data to high-dimensional separable data (Cortes and Vapnik, 1995). SVM with a linear kernel was used to reduce the risk of overfitting the training data and directly extract the feature weights (Pereira et al., 2009). We utilized “leave-one-out” cross-validation method to provide an unbiased estimation of the performance of aforementioned features. Specifically, in each cross-validation experiment, one participant was selected as the test subject, and the rest 58 participants were viewed as the training set. The inter-group differences in frequency-specific S-FCD/L-FCD within the training set were detected by conducting two-sample t-test and the mean S-FCD/L-FCD values of the voxels in the inter-group difference clusters were also calculated as classification features. We repeated this procedure 59 times. Moreover, the receiver operating characteristic (ROC) curves and the area under the curves (AUC) were used to evaluate whether FCD with significant between-group differences could be utilized as markers to discriminate the GAD patients from the HCs. All the above classification analysis were conducted by the LIBSVM 3.22 matlab toolbox^4^. An overview of the proposed classification procedure with frequency-specific FCD framework was summarized in **Figure 1**.

## Results

### Frequency Distribution of IMF in GAD and HC

**FIGURE 2:**
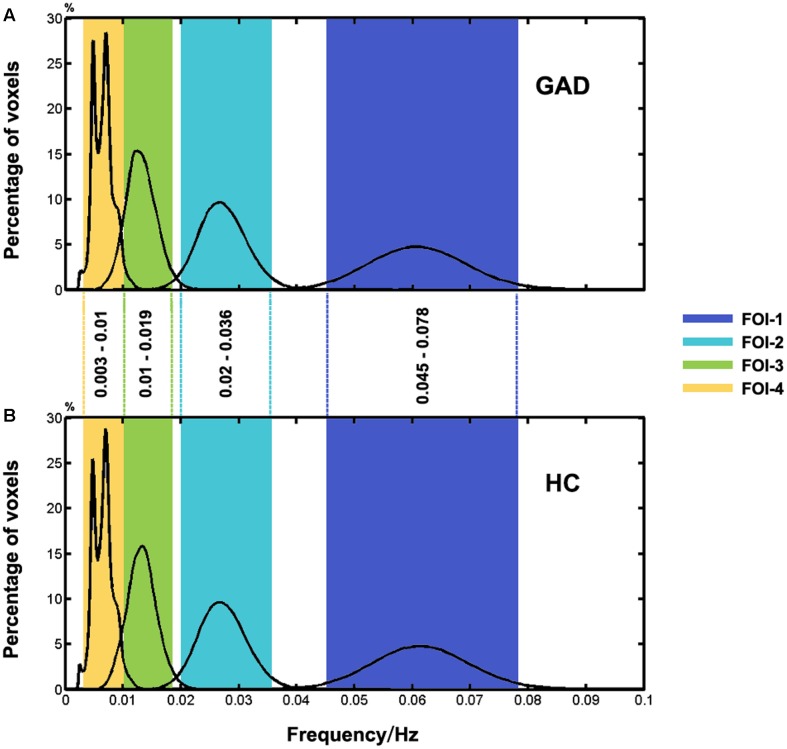
Histogram of frequency distribution between two groups. The histograms of the HWF distributions show the first four intrinsic mode functions (IMFs) of each voxel in the whole-brain gray matter across all participants in each group by using the CEEMDAN approach. Each of the sub-histograms in corresponding IMFs displays amount of the whole-brain gray matter voxels within the GAD **(A)** and HC **(B)** group, respectively. Vertical axis represents the percentage of the number of voxels with HWF equal to the frequency on the horizontal axis in the whole-brain gray matter. The rectangular bar with different colors represents the frequency bands of the final selected FOIs. Colors assigned in the sequence of blue, cyan, green, and yellow represent FOI-1 (0.045–0.078 Hz), FOI-2 (0.02–0.036Hz), FOI-3 (0.01–0.019 Hz), and FOI-4 (0.003–0.01 Hz), respectively.

As shown in **Figure 2**, four FOIs intervals (0.045–0.078 Hz, 0.02–0.036 Hz, 0.01–0.019 Hz, and 0.003–0.01 Hz) were finally selected to ensure the consistency of frequency bands between the two groups. For simplicity, we numbered them from FOI-1 to FOI-4, where FOI-1 was the highest frequency interval and FOI-4 was the lowest. We named these four intervals together as FOI-S (including FOI-1 to FOI-4) when compared with FOI-N.

### Frequency-Specific FCD Alterations in GAD

**FIGURE 3:**
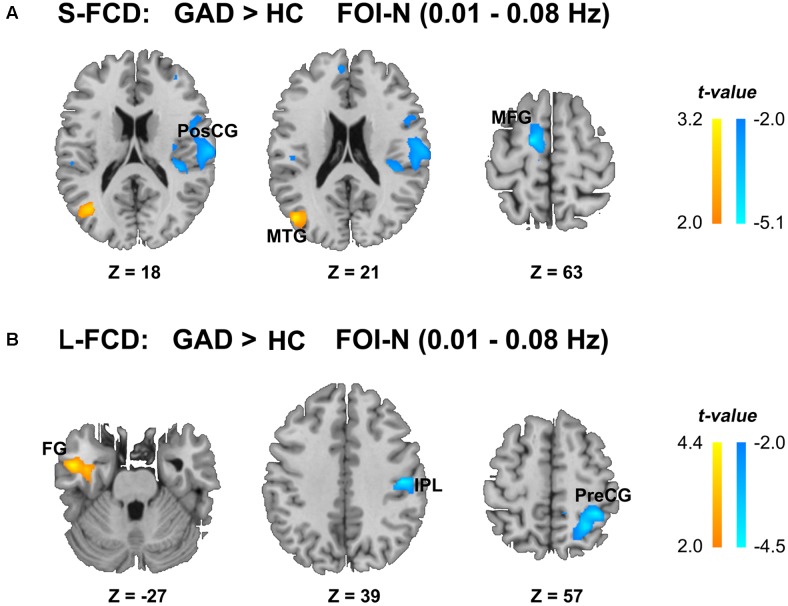
Statistical differences of FCD between two groups in the conventional frequency band FOI-N (0.01–0.08 Hz). **(A)** Statistical differences of S-FCD between two groups. **(B)** Statistical differences of L-FCD between two groups. The color bar indicates the t values, which represent increased (warm) and decreased (cool) S-FCD and L-FCD in the GAD patients compared with HCs. Two sample *t*-test, significant level was set at *P* < 0.05, with AlphaSim corrected. MTG, middle temporal gyrus; PosCG, postcentral gyrus; MFG, middle frontal gyrus; FG, fusiform gyrus; IPL, inferior parietal lobule; PreCG, precentral gyrus. The left hemisphere is on the left.

We observed between-group differences of the S-FCD and L-FCD in conventional frequency band FOI-N (*P* < 0.05, AlphaSim corrected, with a cluster size of 220 voxels). Compared to the HC, S-FCD of GAD patients significantly increased in the left middle temporal gyrus (MTG), but decreased in the right postcentral gyrus (PosCG) and left middle frontal gyrus (MFG). Meanwhile, L-FCD in GAD patients increased in the left fusiform gyrus (FG), but decreased in the right precentral gyrus (PreCG) and right inferior parietal lobule (IPL) (see **Table 2** and **Figure 3**).

**Table 2:** Brain areas showing significant differences in FCD between patients with GAD and HCs in FOI-N (0.01–0.08 Hz).

Anatomical regions	BA	MNI coordinates			Cluster size	Maximal *t*-value
				
		*X*	*Y*	*Z*		
S-FCD						
GAD < NC						
Right postcentral gyrus (PosCG)	4	63	–15	18	599	–3.99
Left middle frontal gyrus (MFG)	6/32	–15	–6	63	265	–5.06
GAD > NC						
Left middle temporal gyrus (MTG)	39	–45	–72	21	254	3.12
L-FCD						
GAD < NC						
Right precentral gyrus (PreCG)	4	45	–18	39	364	–3.56
Right inferior parietal lobule (IPL)	40	36	–42	57	252	–4.52
GAD > NC						
Left fusiform gyrus (FG)	20	–51	–6	–27	246	4.40


*Maximal *t*-value, statistical value of peak voxel showing S-FCD/L-FCD differences between two groups. BA, Brodmann’s area; MNI, Montreal Neurological Institute; X/Y/Z, coordinates of peak locations in the MNI space. *P* < 0.05, corrected for AlphaSim.*

**FIGURE 4:**
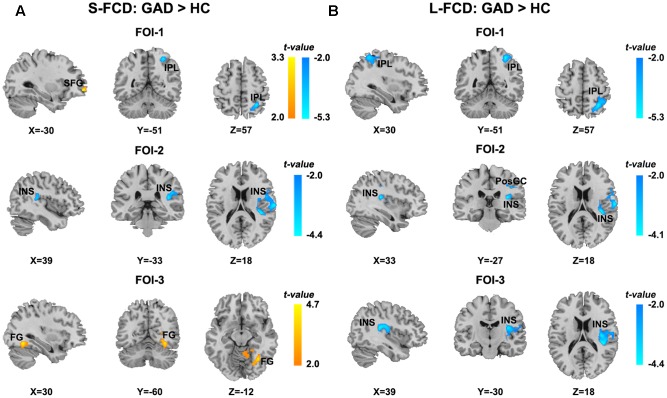
Statistical differences of frequency-specific FCD between two groups. **(A)** Statistical differences of S-FCD between two groups in three frequency-specific bands. **(B)** Statistical differences of L-FCD between two groups in three frequency-specific bands. The color bar indicates the *t-*values, which represent increased (warm) and decreased (cool) S-FCD and L-FCD in the GAD patients compared with HCs. Two sample *t*-test, significant level was set at *P* < 0.05, with AlphaSim corrected. The left hemisphere is on the left.

Significant between-group differences of the S-FCD and L-FCD were also found in three frequency-specific bands. Compared to the HC, GAD patients showed significant frequency-specific S-FCD and L-FCD alterations (*P* < 0.05, AlphaSim corrected, with a cluster size of 220 voxels, see **Table 3** and **Figure 4**). Specifically, S-FCD of GAD patients in the left superior frontal gyrus (SFG) (FOI-1) and right FG (FOI-3) significantly increased, but decreased in the right IPL (FOI-1) and right insula (INS) (FOI-2). Further, L-FCD of GAD patients decreased in the right IPL (FOI-1), right PosCG (FOI-2), and right INS (FOI-2 and FOI-3). No significant between-group difference was found in frequency band FOI-4.

**Table 3:** Brain areas showing significant differences in frequency specific FCD between patients with GAD and HCs.

Anatomical regions	BA	MNI coordinates			Cluster size	Maximal *t*-value
				
		*X*	*Y*	*Z*		
S-FCD						
FOI-1						
GAD < HC						
Right inferior parietal lobule (IPL)	7	30	–51	57	242	–4.12
GAD > HC						
Left superior frontal gyrus (SFG)	10	–30	57	–3	223	3.17
FOI-2						
GAD < HC						
Right insula (INS)	13	39	–33	18	599	–4.37
FOI-3						
GAD > HC						
Right fusiform gyrus (FG)	18	30	–60	–12	466	4.67
L-FCD						
FOI-1						
GAD < HC						
Right inferior parietal lobule (IPL)	7/40	30	–51	57	284	–5.29
FOI-2						
GAD < HC						
Right postcentral gyrus (PosCG)	3/4	63	–15	18	294	–3.84
Right insula (INS)	13	33	–27	18	266	–4.13
FOI-3						
GAD < HC						
Right insula (INS)	13	39	–30	18	461	–4.38


*Maximal *t*-value, statistical value of peak voxel showing S-FCD/L-FCD differences between two groups. BA, Brod mann’s area; MNI, Montreal Neurological Institute. *X/Y/Z*, coordinates of peak locations in the MNI space. *P* < 0.05, corrected for AlphaSim.*

### Correlation Analyses

**FIGURE 5:**
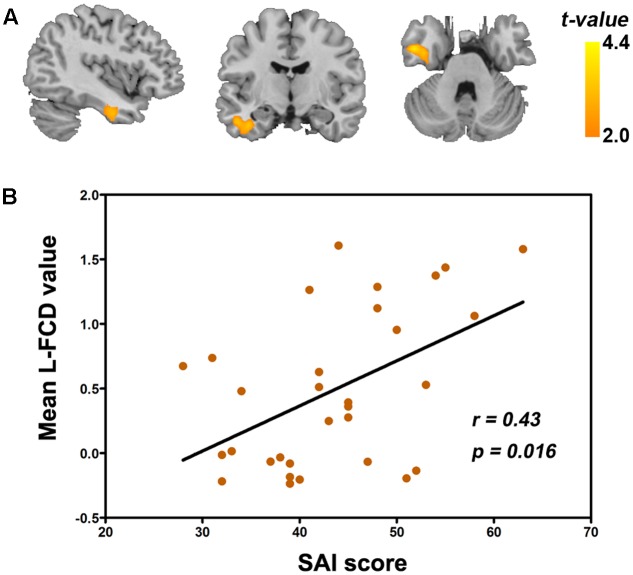
Correlation between the state anxiety inventory (SAI) score and L-FCD in patients with GAD. **(A)** The positive correlation between the SAI score and L-FCD of the left FG. **(B)** The scatter plot of the correlation in this region. The color bar represents the statistical *t*-values. *P* < 0.05, corrected for FDR.

As seen in **Figure 5**, within FOI-N, state anxiety inventory (SAI) score was positively correlated with L-FCD of the left FG in GAD (*r* = 0.43, *P* = 0.016, FDR corrected). We also computed the correlation, respectively, between clinical scales and S-FCD/L-FCD in all frequency-specific regions that showed significant between-group differences, but no significant correlation was found (*P* > 0.05).

### Classification Performance

**FIGURE 6:**
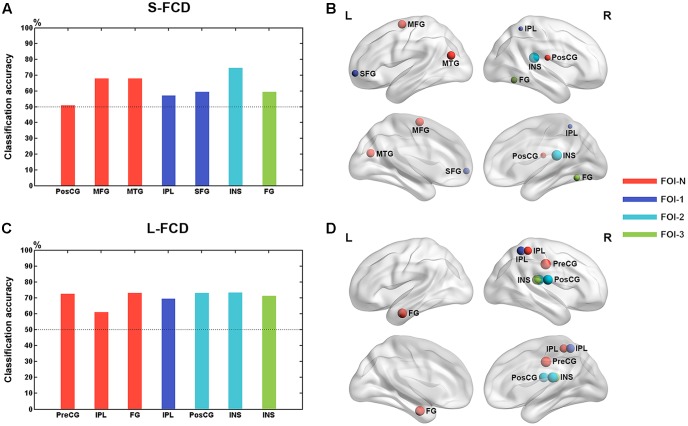
Classification performance of each between-group difference region. The classification accuracy of each between-group difference region in S-FCD **(A)** and L-FCD **(C)**. Rendering plot of the between-group difference regions in S-FCD **(B)** and L-FCD **(D)** used in the classification. Colors assigned in the sequence of red, blue, cyan, and green represent the frequency bands such as FOI-N, FOI-1, FOI-2, and FOI-3, respectively. The size of the node in **(A,B)** represents the accuracy of the normalized region classification performance. The left hemisphere is on the left.

The mean values of frequency-specific S-FCD and L-FCD with between-group difference within each cluster were extracted for discriminating GAD patients from HCs. We observed the classification performance when considering the S-FCD/L-FCD of between-group difference region at each frequency band and the classification accuracy peaked when using the right INS of FOI-2 (74.65%) (see **Figure 6**). The detailed classification performance of each region in each frequency band was presented in Supplementary Figures and Tables.

**FIGURE 7:**
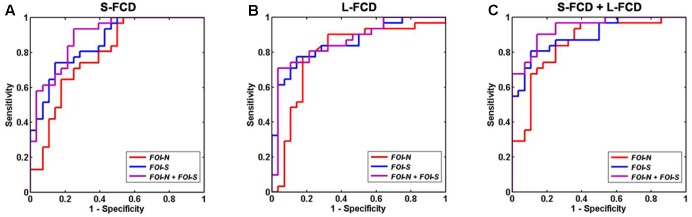
Receiver operating characteristic (ROC) of discriminating GAD patients from HCs by using FCD values in the significant group difference regions. **(A)** S-FCD values in the significantly different regions; **(B)** L-FCD values in the significantly different regions; **(C)** a combination of S-FCD and L-FCD values in the significantly different regions. In **(A–C)**, red, blue, and purple line represent the FCD in FOI-N, FOI-S, and FOI-N+FOI-S, respectively.

The classification performance of using discrepant S-FCD and L-FCD from FOI-N, FOI-S, and the combination of FOI-N and FOI-S were further examined. Results of S-FCD separately received the AUC values of 0.6117, 0.7503, and 0.9002 with corresponding classification accuracies of 72.94, 74.61, and 83.43% (**Table 4** and **Figure 7A**). Compared to S-FCD, the L-FCD showed better classification performance which separately achieved the AUC values of 0.7626, 0.8547, and 0.8633 with corresponding accuracies of 81.42, 83.11, and 84.53%, respectively (**Table 4** and **Figure 7B**). The classification performance were further improved by combining both S-FCD and L-FCD, as the AUC values of 0.8731, 0.8993, and 0.9414 were, respectively, obtained with corresponding classification accuracies of 84.75, 85.74, and 89.83% (**Table 4** and **Figure 7C**).

**Table 4:** Discriminating the GAD participants from the HCs by receiver operating characteristic (ROC) analyses.

Brain regions	AUC	Accuracy (%)	Sensitivity (%)	Specificity (%)
S-FCD				
FOI-N	0.6617	72.94	80.72	64.37
FOI-S	0.7503	74.61	71.03	78.60
FOI-N + FOI-S	0.9002	83.43	87.11	79.55
L-FCD				
FOI-N	0.7626	81.42	90.71	71.98
FOI-S	0.8547	83.11	83.90	82.25
FOI-N + FOI-S	0.8633	84.53	83.44	85.73
S-FCD + L-FCD				
FOI-N	0.8731	84.75	87.13	82.17
FOI-S	0.8993	85.74	82.47	89.11
FOI-N + FOI-S	0.9414	89.83	87.15	92.92


## Discussion

In the current study, frequency-specific FCD analysis was carried out to explore aberrant SFBO in adolescent patients with GAD. Our findings showed that GAD patients had significant differences in S-FCD and L-FCD across different frequency bands compared with HCs. Furthermore, significantly positive correlation between the SAI and L-FCD in the left FG was found in the conventional frequency band FOI-N (0.01–0.08 Hz). More importantly, the right insula was found to have the highest classification accuracy in the lower frequency band FOI-2 (0.02–0.036 Hz) in discriminating GAD patients from control participants compared with all other between-group difference regions and a satisfactory classification performance was achieved by combining the discrepant S-FCD and L-FCD values in all frequency bands. Our findings indicated that abnormal FCD in some brain regions in GAD patients was frequency dependent and frequency-specific FCD could be used in the diagnosis of an individual patient with GAD.

In the conventional frequency band FOI-N, brain regions with abnormal S-FCD in GAD patients were mainly located in the left MTG, left PosCG and left MFG, whereas brain regions with abnormal L-FCD were mainly located in the right PreCG, right IPL and left FG, showing disparately spatial patterns of the S-FCD versus L-FCD. The MTG is associated with recognition of known faces and accessing word meaning while reading (Acheson and Hagoort, 2013). Increased S-FCD in MTG may reflect that GAD patients show enhanced attention toward negative information in comparison to HCs. Moreover, the PreCG and PosCG, which have been reported to be involved in interoception processing (Northoff, 2013), displayed reduced S-FCD and L-FCD in GAD patients, respectively. A task-based fMRI study has depicted higher activities in PreCG in response to the anxiety-inducing words in GAD patients (Moon et al., 2015). Our results indicates disruption of FCD in PreCG and PosCG may induce abnormality in integration of network and mutual adjustment between cognitive function in most cases (Di Bono et al., 2017). Additionally, abnormal FCD in the MFG, IPL and FG were also found, and these regions have been involved in various of cognitive processes (Yang and Raine, 2009). Long-term existence of excessive and uncontrollable fear and worry in GAD patients might be thought to result in disruption of intrinsic cognitive function organization. Different from previous amygdala-based FC studies, our results showed that widespread changes of SFBO existed in adolescent GAD.

Notably, the GAD patients showed significantly positive correlation between increased L-FCD in the left FG and the SAI score that evaluates a kind of transient emotional state provoked by risky stimulus, including individual stress, worry, anxiety, distress, and excessive excitement of the autonomic nerve system (Spielberger et al., 1970). The FG, part of the temporal lobe and occipital lobe, has been indicated to serve a vital function in recognizing face, body, and words (Haxby et al., 2002; Grill-Spector and Malach, 2004; Martin, 2007). Previous studies reported that the anxiety disorder patients possessed of abnormal activities in the FG when participating in the task related to emotional face, suggesting that FG might play an important role in emotion and visual processing (Gentili et al., 2008; Syal et al., 2012). Furthermore, resting-state fMRI study on social anxiety disorder patients also showed significantly increased FCstrength in the fusiform gyrus compared to HCs (Liu et al., 2015b). Specially, FG is usually functional connected with the hippocampus which belongs to the limbic system and it involves fear generalization processing (Xu and Sudhof, 2013). In a recent resting-state FC study in GAD, the researchers reported that the increased FC between hippocampus and FG indicated that abnormal SFBO was mainly related to fear generalization associated with neural circuit and was responsible for the emotion dysregulation in GAD (Cui et al., 2016). In our study, the increased L-FCD in the left FG might be associated with the dysfunction of transient emotional processing, which might help to improve the emotional regulation abilities of adolescent GAD patients.

In three frequency-specific bands, GAD patients showed altered S-FCD and L-FCD primarily distributed over the right IPL, left SFG, right INS, right FG, and right PosCG, reflecting incomplete consistent inter-group difference regions compared with FOI-N. Both IPL and INS belong to the default mode network (DMN), which is the most well-characterized intrinsic connectivity network and its disruption can result in various emotional dysfunctions (Akansha et al., 2016). GAD patients always show deficiency in emotion processing such as limited self-control, concentrating difficulty, and response inhibition (Fonzo and Etkin, 2016). Our findings are in line with the perspective that emotional dysregulation derived from disrupted DMN is an universal phenomenon in GAD (Mochcovitch et al., 2014). Interestingly, both S-FCD and L-FCD in GAD patients were reduced in the right IPL at higher frequency band FOI-1 but showed no significant alterations at lower frequency bands such as FOI-2 and FOI-3, and reduced in the right INS at lower frequency bands FOI-2 but did not significantly change in FOI-1, which indicated that the alterations of FCD were frequency dependence in GAD patients and the frequency band FOI-1might be more sensitive to detect abnormal SFBO in right IPL than others, whereas the frequency band FOI-2 might be more sensitive to detect abnormal SFBO in right INS than others. Previous studies have observed frequency dependent changes of intrinsic brain fluctuations in resting-state in other diseases, suggesting that intrinsic brain activities are sensitive to specific frequency bands. For example, a resting-state study in social anxiety disorder has showed that ALFF reduced in the 0.01–0.027 Hz but did not change in the 0.027–0.073 Hz (Zhang et al., 2015). Another study suggested that the alterations of low-frequency oscillation amplitudes in specific brain regions in major depressive disorder patients could be more sensitively detected in the 0.01–0.027 Hz rather than the 0.027–0.073 Hz bands (Wang L. et al., 2016). Since brain fluctuations of different frequencies may have different physiological implications, the GAD-related abnormal FCD in distinct brain regions may have different sensitivity toward different frequency bands. Moreover, increased S-FCD in the left SFG (FOI-1) and in the right FG (FOI-3), and reduced L-FCD in the right PosCG (FOI-2) in GAD patients were also found in the present work. These findings also suggested that widespread brain regions were presented in frequency-specific disruptions and diverse sensitivity in detecting alterations of SFBO might rely on choosing frequency bands in adolescent GAD patients.

We further observed the classification performance of the FCD value in sole between-group difference region when discriminating GAD patients from HCs. We found that the insula in a lower frequency band FOI-2 had the optimal classification performance with the highest classification accuracy reaching 74.65%, which was an acceptable accuracy for established diagnostic indicators (Swets, 1988). As mentioned above, decreased FCD in INS might reflected a disruption DMN in emotional dysregulation for GAD patients. Additionally, evidence from other perspective also demonstrated abnormal activity in INS during emotion regulation process in GAD patients. For example, task-based fMRI studies have shown the hyperactivity of insula in response to negative emotion in anxious individuals (Hoehn-Saric et al., 2004; Gentili et al., 2008; Etkin et al., 2011) and decreased activity when using anxious words stimuli after seven weeks of citalopram treatment (Paulus and Stein, 2010), which indicates that insula activity may be a key neural mechanism underlying anxiety-related processes. The insula is also thought to enable to perceive the information of salience, emotion, as well as attention and then integrate and send such information to amygdala (Nieuwenhuys, 2012; Baur et al., 2013), which has been found to be structurally connected to the insula [60]. Recent studies suggest that these processes are disrupted in adolescent patients with GAD who exhibited abnormal FC between the amygdala and the insula both in resting-state (Etkin et al., 2009; Roy et al., 2013; Hamm et al., 2014) and task-state (Hoehn-Saric et al., 2004; Moon et al., 2015), which possibly leads to emotional dysregulation due to a neglect of some typical information in interoceptive stimuli from potentially aversive body signals (Paulus and Stein, 2010). GAD adolescents often have uncontrollable fear and worry, possibly because the reduced insula FCD disrupted the function in integrating information transferred from the insula to the amygdala. Moreover, Mcclure et al. (2007) discovered a positive correlation between the scores in a state of anxiety and amygdala-insula FC, which was considered to be a biological marker of anxiety. Our results were in line with the previous findings that abnormal insula activity was ubiquitous in GAD and suggested that the specific symptomatology of GAD might be related to the abnormal insula activity in the lower frequency band (0.02–0.036 Hz). These also implied that the FCD in the insula in the lower frequency band could be as a potential biomarker to discriminate adolescent patients with GAD from HCs. Further study should pay more attention to investigate the pathophysiological mechanisms of insula in GAD.

In addition, the ROC results indicated that the L-FCD has better classification performance for discriminating the GAD patients from the HCs than S-FCD when combining group difference regions in three different frequency band conditions, reflecting that alteration of long-range functional connections is a more obvious biomarker in GAD. Previous study reported that only aberrant L-FCD was found in post-traumatic stress disorder (PSD) (Zhang et al., 2016), indicating neurodegenerative disruption of intrinsic oscillations and connectivity in PSD mainly affected long-distance connections (Liu et al., 2014). Our results showed that frequent worry in GAD patients might more result from the disruptions in long-distance connections which were associated with the consequent loss of brain network efficiency. Finally, the combination of S-FCD and L-FCD in all frequency-specific difference regions was used to discriminate GAD patients from HCs and the optimum classification performance achieved that the AUC value was 0.9414 and its corresponding classification accuracy was 89.83%. Recently, a multimodal machine learning study on separating GAD and major depression from HCs achieved 90.10% accuracy by combining the data in clinical, cortisol, and structural MRI (Hilbert et al., 2017). To our knowledge, there is few study discriminating GAD patients from HCs adopting resting-state FC approach, but results from a research on social anxiety disorder by using whole-brain FC have showed a correct classification rate of 82.5% (Liu et al., 2015a). Compared with previous studies, our frequency-specific FCD approach had a satisfactory classification performance and we thus inferred that frequency-specific S-FCD and L-FCD could be employed as an effective means to diagnose GAD.

However, several limitations were noteworthy. First, the sample data was limited in the current study, so large sample is needed in future studies. Second, we used a relatively low sampling rate (TR = 3000 ms) for multiple (36 slices) acquisitions in our study. Although this sampling rate helped to minimize some physiological noises such as cardiac fluctuations and respiratory, physiological noises could not be removed entirely. Thus, future research should try to apply a more rigorous data analysis method to remove noises. Third, we observed the SFBO in a low frequency band (<0.08 Hz) but ignored the high frequency band (>0.08 Hz) that is always thought to include some respiratory signals at the frequency range from 0.1 to 0.5 Hz (Cordes et al., 2001). Recently, several studies extended the observation of the pattern of intrinsic brain activity to a high frequency band (>0.1 Hz), which demonstrated consistent patterns with low frequency fluctuations (<0.1 Hz) (Boubela et al., 2013), indicating that some valuable physiological information might be included in the high frequency band. Finally, we only selected a single threshold to calculate FCD maps, so a range of thresholds can be tried to test the stability of the results in future study.

## Conclusion

We used the frequency-specific FCD mappings to investigate the SFBO of the adolescent GAD patients. The results showed that GAD patients exhibited abnormal S-FCD and L-FCD in widespread brain regions across different frequency bands, and a satisfactory classification performance was gained in discriminating GAD patients from HCs by using abnormal frequency-specific FCD values. Thus, it seems possible that frequency-specific FCD can be used as an effective biomarker in diagnosing GAD in the future. The findings highlight the importance of frequency specificity in SFBO and will deepen our understanding of the pathophysiological mechanism underlying GAD.

## Author Contributions

ZZ and ML contributed equally to this work. ML and LL designed and performed the experiments. ZZ, YX, and ZY researched data, contributed to discussion, and wrote, reviewed, and edited the manuscript. ML, BH, and WZ researched data, contributed to discussion, and reviewed and edited the manuscript. TH, YZ, FY, YZ, LS, LL, JG, and DM contributed to discussion, and reviewed and edited the manuscript.

## Conflict of Interest Statement

The authors declare that the research was conducted in the absence of any commercial or financial relationships that could be construed as a potential conflict of interest.

## Footnotes

1 http://www.restfmri.net

2 http://www.fil.ion.ucl.ac.uk/spm

3 http://rfmri.org/dpabi

4 http://www.csie.ntu.edu.tw/~cjlin/libsvm/
