# Supplementary material for: Frequency-Specific Functional Connectivity Density as an Effective Biomarker for Adolescent Generalized Anxiety Disorder
Source: Front Hum Neurosci. 2017 Dec 5;11:549. doi: 10.3389/fnhum.2017.00549 (PMC5723402; doi:10.3389/fnhum.2017.00549)
Supplement: Supplementary file 1 [file Data_Sheet_1.pdf]

Supplementary Material

Frequency-specific functional connectivity density as an effective biomarker for adolescent generalized anxiety disorder

Zhe Zhang, Mei Liao, Zhijun Yao, Bin Hu*, Yuanwei Xie, Weihao Zheng, Tao Hu, Yu Zhao, Fan Yang, Yan Zhang, Linyan Su, Lingjiang Li*, Jürg Gutknecht, Dennis Majoe

*** Correspondence:** Bin Hu: bh@lzu.edu.cn; Lingjiang Li: llj2920@163.com

1 Supplementary Figures

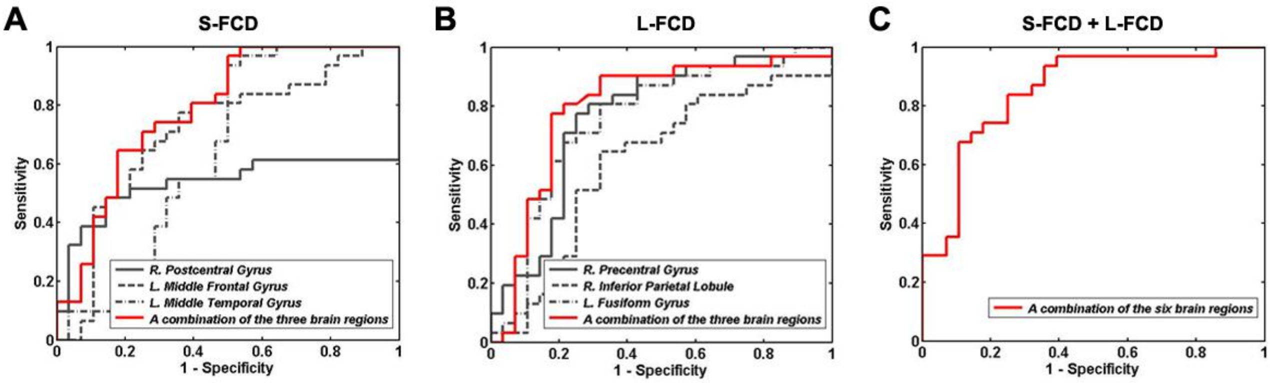

Supplementary Figure 1. ROC of discriminating GAD patients from HCs by using FCD values of the significant group difference regions in FOI-N. (A) S-FCD values in the significantly different regions; (B) L-FCD values in the significantly different regions; (C) a combination of S-FCD and L-FCD values in the significantly different regions.

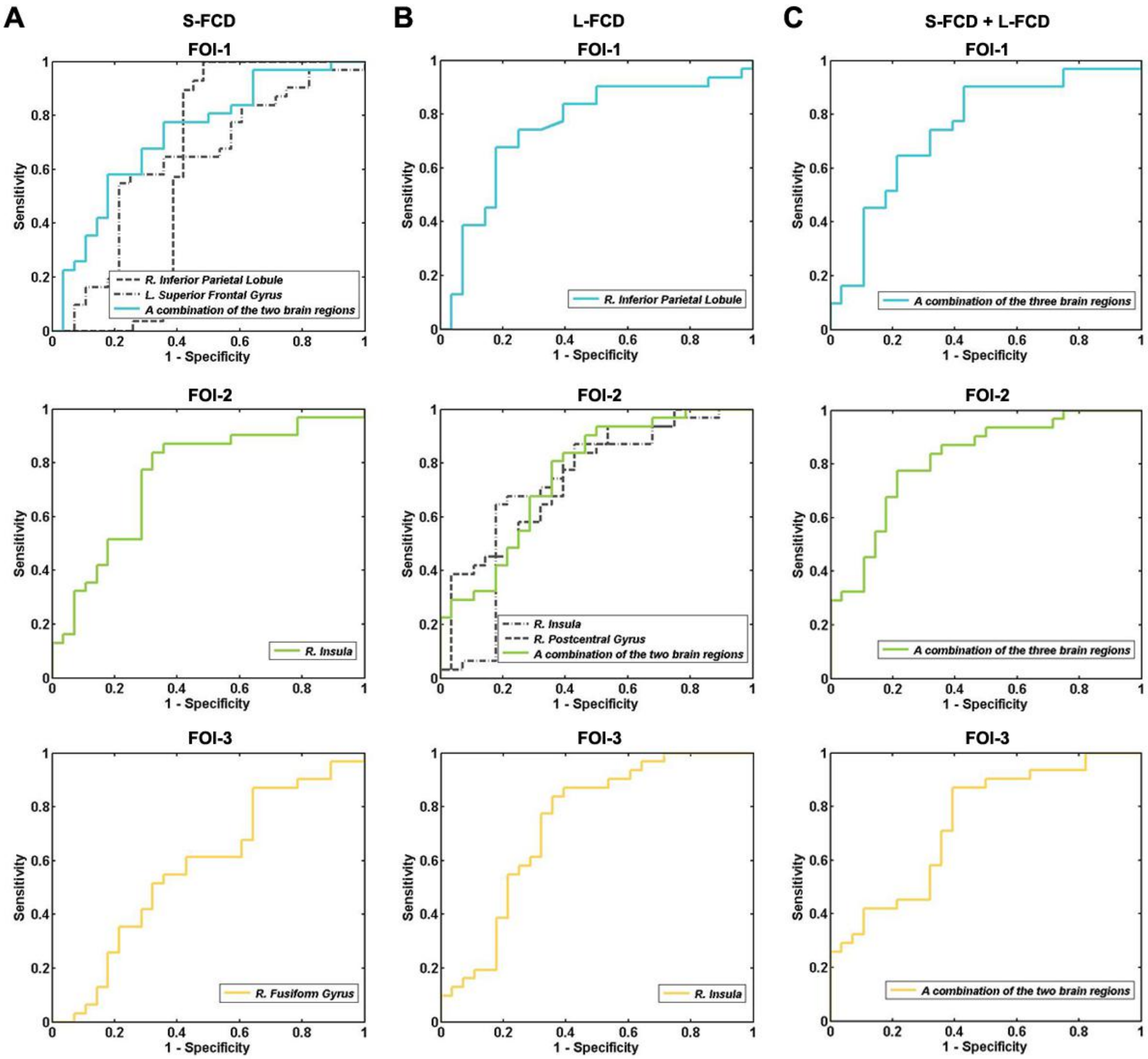

Supplementary Figure 2. ROC of discriminating GAD patients from HCs by using FCD values of the significant group difference regions in FOI-S. (A) S-FCD values in the significantly different regions; (B) L-FCD values in the significantly different regions; (C) a combination of S-FCD and L-FCD values in the significantly different regions.

2 Supplementary Tables

Supplementary Table 1. Discriminating the GAD patients from the healthy controls by ROC analyses in FOI-N.

Brain regions	AUC	Accuracy	Sensitivity	Specificity
S-FCD				
Right postcentral gyrus	0.5334	50.82%	54.82%	46.47%
Left middle frontal gyrus	0.7123	67.87%	71.03%	64.32%
Left middle temporal gyrus	0.6394	67.87%	83.97%	50.04%
A combination of the three brain regions	0.7886	67.87%	74.25%	60.72%
L-FCD				
Right precentral gyrus	0.7624	72.63%	81.93%	62.32%
Right inferior parietal lobule	0.6111	61.04%	74.22%	46.43%
Left fusiform gyrus	0.7553	72.92%	71.04%	75.04%
A combination of the three brain regions	0.7975	78.07%	83.99%	72.45%

Supplementary Table 2. Discriminating the GAD patients from the healthy controls by ROC analyses in FOI-S.

Brain regions	AUC	Accuracy	Sensitivity	Specificity
S-FCD				
FOI-1				
Right inferior parietal lobule	0.6043	57.16%	62.74%	51.63%
Left superior frontal gyrus	0.6295	59.33%	74.25%	42.95%
A combination of the two brain regions	0.7337	67.85%	71.04%	64.32%
FOI-2				
Right insula	0.7504	74.65%	83.93%	64.33%
FOI-3				
Right fusiform gyrus	0.5742	59.31%	61.37%	57.17%
L-FCD				
FOI-1				
Right inferior parietal lobule	0.7514	69.56%	77.42%	60.73%
FOI-2				
Right postcentral gyrus	0.7178	72.97%	83.95%	61.78%
Right insula	0.7515	73.23%	83.93%	62.23%
A combination of the two brain regions	0.7583	71.24%	83.92%	58.18%
FOI-3				
Right insula	0.7399	71.27%	77.44%	64.33%
S-FCD + L-FCD				
FOI-1				
A combination of the three brain regions	0.7484	69.55%	74.22%	64.34%
FOI-2				
A combination of the three brain regions	0.8173	74.62%	83.97%	64.32%
FOI-3				
A combination of the two brain regions	0.7397	69.54%	77.43%	60.78%
